# The Role of DJ-1 in the Pathogenesis of Endometriosis

**DOI:** 10.1371/journal.pone.0018074

**Published:** 2011-03-21

**Authors:** Priyanka Rai, Sisinthy Shivaji

**Affiliations:** Centre for Cellular and Molecular Biology, Hyderabad, India; Institut de Génomique Fonctionnelle de Lyon, France

## Abstract

**Background:**

Endometriosis is an estrogen-dependent disease causing pelvic pain and infertility in 10% of reproductive-aged women. Despite a long history of the disease the pathogenesis of endometriosis is poorly understood. It is known that the expression of several proteins is either up or down regulated during endometriosis, but their precise role remains to be determined. DJ-1 is one such protein that is upregulated in eutopic endometrium of women having endometriosis suggesting that DJ-1 may be involved in the pathogenesis of endometriosis.

**Methodology and Principal Findings:**

The role of DJ-1 in the pathogenesis of endometriosis was investigated. For this purpose the influence of DJ-1 on endometrial cell survival, attachment, proliferation, migration, and invasion either by overexpressing DJ-1 in normal endometrial cells or by knocking down DJ-1 expression in endometriotic cells using siRNA was investigated. The results indicated that DJ-1 protects endometrial cells from oxidative stress mediated apoptosis. Overexpression of DJ-1 in normal endometrial epithelial cells increases the adhesion on collagen type IV. However, no significant difference was observed incase of stromal cells. It was further demonstrated that DJ-1 regulates cell proliferation, migration, and invasion in normal endometrial and endometriotic epithelial cells whereas in the case of normal endometrial and endometriotic stromal cells, it regulates cell proliferation and invasion but not migration. Furthermore, the present study also indicated that DJ-1 regulates these cellular processes by modulating PI3K/Akt pathway by interacting and negatively regulating PTEN.

**Conclusions:**

Abnormally high levels of DJ-1 expression may be involved in endometriosis, possibly by stimulating endometrial cell survival, proliferation, migration, and invasion.

## Introduction

Endometriosis is a complex gynecological disease which occurs in 10% of reproductive age women. The disease is characterized by the presence and growth of endometrial tissue outside the uterus, causing pelvic pain, and infertility [1]. The pathogenesis of endometriosis is not clearly defined. However, the disease is thought to be principally caused by the shedding of viable endometrial cells into the peritoneal cavity by retrograde menstruation, followed by their implantation and growth on the surface of pelvic organs [1]. The formation of a lesion depends on the survival, attachment, growth, neoangiogenesis, and invasion of the endometrial cells at the ectopic sites [2]. This may be due to abnormalities of the eutopic endometrium itself, predisposing the cells to survive and implant ectopically [3].

Several studies have shown aberrant expression of genes/proteins in endometriosis that are involved in regulating cellular processes like adhesion, proliferation, angiogenesis, immune dysfunction, and others [4]–[9]. Recently, using proteomics approach, we have investigated the differential expression of proteins in eutopic endometrium from women with and without endometriosis [9]. In this study it was observed that DJ-1 protein is upregulated in eutopic endometrium of women having endometriosis compared with controls. These findings suggest that DJ-1 may be involved in the pathogenesis of endometriosis.

The human DJ-1 gene comprises of seven exons and maps to 1p36.2–36.3, where many chromosome aberrations in cancers have been reported [10]. DJ-1 is ubiquitously present in cells and has been suggested to be a novel mitogen-dependent oncogene involved in a Ras-related signal transduction pathway [11]. More recently, high DJ-1 levels have been reported in various tumors, suggesting that abnormally expressed DJ-1 may play a role in cancer initiation and/or progression under certain circumstances [12]–[16] and may be a potential anticancer target [12]–[15]. DJ-1 protein affects cell survival, proliferation, and growth of cells in part, by modulating cellular signaling cascades such as PTEN-PI3K/Akt [16] and altering p53 activity [17], [18]. DJ-1 has shown to convey protection against stresses (including oxidative stress, and endoplasmic reticulum stress) and proteasome inhibition [12], [19], [20]. It has been suggested that DJ-1 plays a role in anti-oxidative stress by eliminating reactive oxygen species and in transcriptional regulation of its target genes [18], [20].

The pathological significance of DJ-1 in endometriosis has not been elucidated. Therefore, we investigated the effect of DJ-1 on normal endometrial as well in endometriotic cell survival, proliferation, motility, and invasion.

## Results

### Expression of DJ-1 in normal and endometriotic cell lines

**Figure 1:**
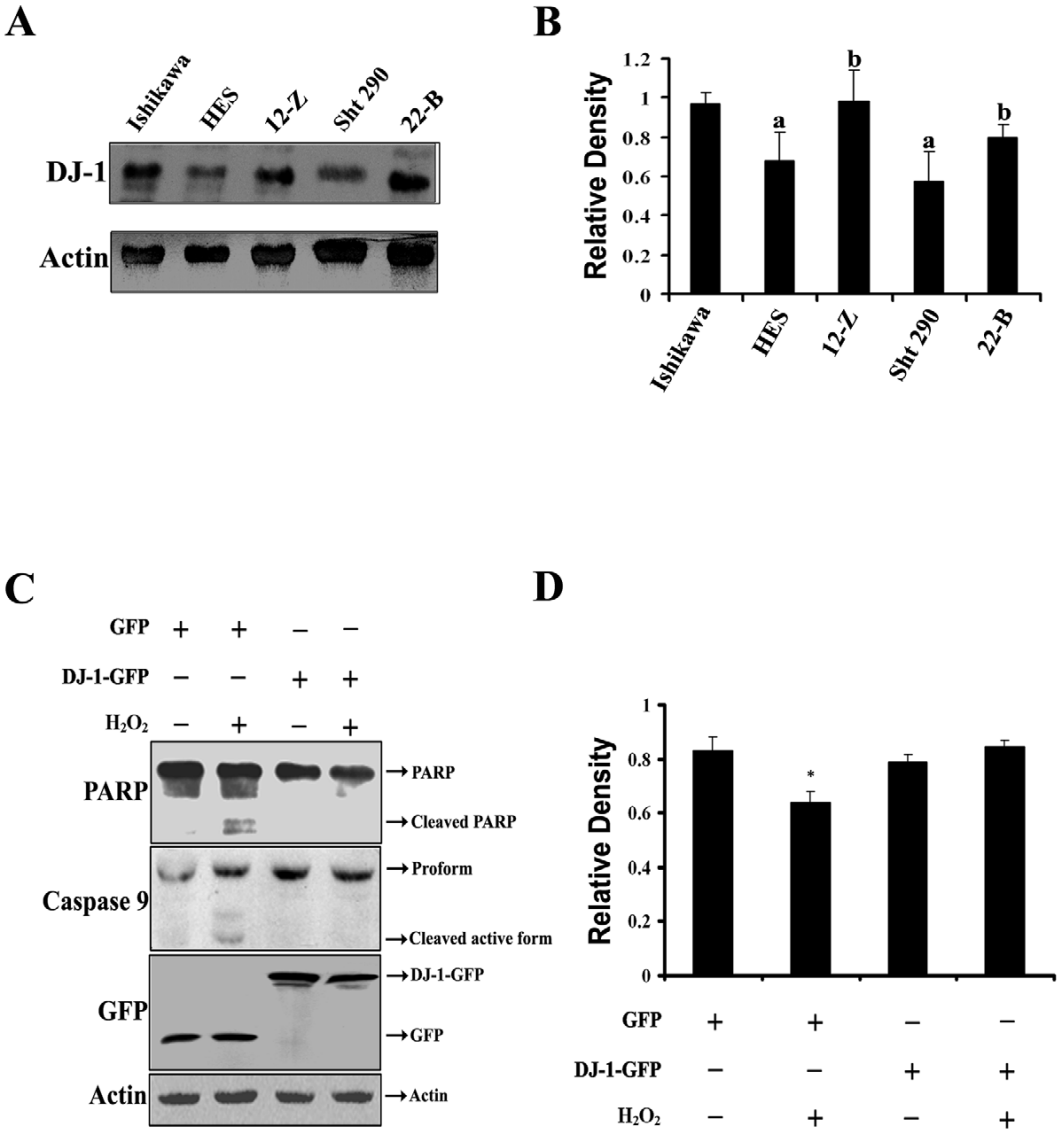
Expression of DJ-1. A, Expression of endogenous DJ-1 in Ishikawa, normal endometrial epithelial (HES) and stromal (Sht 290) cells and endometriotic epithelial (12-Z) and stromal (22-B) cells. B, Band density of DJ-1 relative to that of actin. Bars with different superscripts (a, b) indicate significant differences (p<0.05) between normal endometrial and endometriotic cells, as determined by one way ANOVA with post hoc Bonferroni test. C, DJ-1 protects against apoptosis induced by oxidative stress as it inhibits the cleavage of PARP and caspase-9 induced by oxidative stress. Cells transfected with DJ-1-GFP or GFP were treated with or without 200 µM H_2_O_2_ for 4 h. Equivalent cell lysates were subjected to immunoblot analysis using antibodies against PARP, caspase-9, GFP, and actin. D, Densitometry analysis of PARP protein to actin ratio based on immunoblot analysis. *Asterisk indicates significant difference (p<0.05, as determined by Student's t-test) between cells transfected with DJ-1-GFP or GFP and treated with or without 200 µM H_2_O_2_ for 4 h.

An analysis of endogenous DJ-1 expression in normal human endometrial epithelial (HES) and stromal (Sht 290) cell lines and endometriotic epithelial (12-Z) and stromal (22-B) cell lines was performed. It was observed that the expression of DJ-1 protein was relatively higher in endometriotic cell lines (12-Z and 22-B) compared to normal endometrial cell lines (HES and Sht 290) (Figure 1A and 1B). Ishikawa, which is an adenocarcinoma cell line, was used to show that the DJ-1 expression levels in endometriotic cells were similar to that in endometrial cancer cells (Figure 1A and 1B).

### DJ-1 protects against oxidative stress induced apoptosis

To investigate whether DJ-1 protects against oxidative stress mediated cell death, HES cells were transiently transfected with DJ-1-GFP or GFP alone. After 48 h, cells were exposed to 200 µM hydrogen peroxide (H_2_O_2_) for 4 h. The cells were then collected, and the cell lysates were subjected to immunoblot analysis. We used the cleavage of the 116 kDa PARP to an 89 kDa fragment as a measure of cells undergoing cell death. As shown in Figure 1C and 1D, the cleaved form of PARP (89 kDa) was detected in cells transfected with GFP (control) and exposed to oxidative stress, whereas it was not detected in cells expressing DJ-1-GFP and exposed to oxidative stress (Figure 1C and 1D). Furthermore, pro-caspase-9 was also processed into the active form (p35) in cells expressing GFP and exposed to oxidative stress but not in cells expressing DJ-1-GFP and exposed to oxidative stress (Figure 1C and 1D).

### Overexpression of DJ-1 leads to increased adhesion of normal endometrial epithelial cell

**Figure 2:**
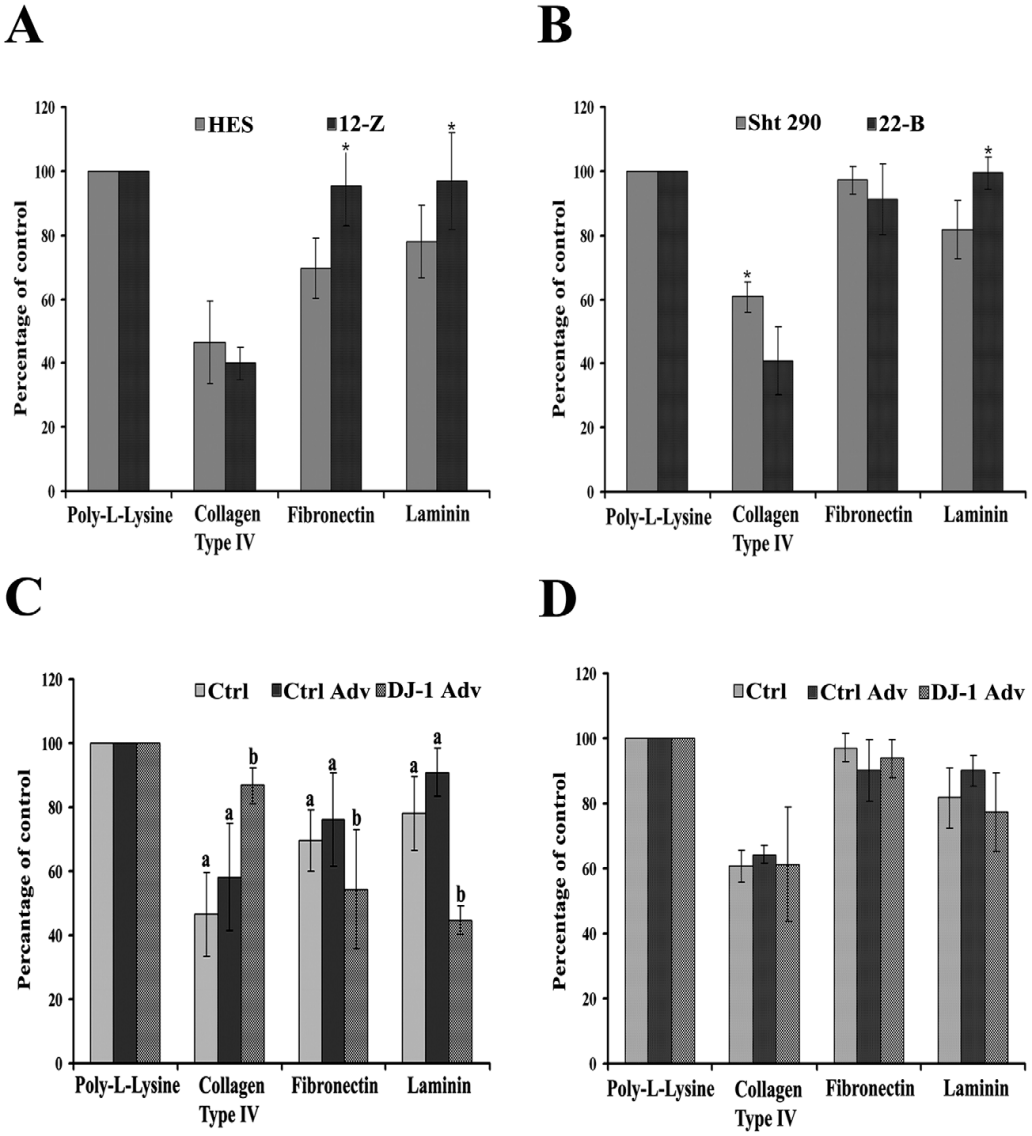
Role of DJ-1 in normal endometrial epithelial and stromal cell attachment. Attachment of normal endometrial epithelial (HES) and endometriotic epithelial (12-Z) cells (A) and normal endometrial stromal (Sht 290) and endometriotic stromal (22-B) cells (B) on various extracellular matrix (ECM) components. C and D depict the effect of DJ-1 overexpression on normal endometrial epithelial (C) and stromal (D) cell attachment. Cells either alone (Ctrl) or infected with control adenovirus (Ctrl Adv) or with DJ-1 adenovirus (DJ-1 Adv) for 24 h were plated on a 96 well plate coated with various ECM components. After 20 min of attachment, MTT assay was performed for determining cell attachment. In figures A and B, * indicates significant difference between HES and 12-Z and Sht 290 and 22-B cells, respectively. In figure C, bars with different superscripts (a, b) indicate significant differences (P<0.05) between cells overexpressing DJ-1 and controls, as determined by one way ANOVA with post hoc Bonferroni test. Numerical data are expressed as mean ± SE of three independent experiments.

Cellular adhesion of normal endometrial and endometriotic cells on various extracellular matrix components (ECM) was performed to understand whether endometriotic cells have the ability to attach at ectopic location. For this, normal endometrial and endometriotic cells were plated on dishes precoated with various ECM components. As shown in Figure 2A, endometriotic epithelial cells (12-Z) attached significantly more on fibronectin and laminin but not on collagen type IV when compared to normal endometrial epithelial cells (HES). Endometriotic stromal cells (22-B) show more attachment on laminin but less on collagen type IV when compared with normal endometrial stromal cells (Figure 2B). However, no significant difference in attachment was observed between normal endometrial and endometriotic stromal cells on fibronectin (Figure 2B).

To determine whether DJ-1 augments cellular adhesion, normal endometrial cells were infected with DJ-1 or control adenovirus for 24 h and cell adhesion assay was performed. It was observed that HES cells overexpressing DJ-1 show increased attachment on collagen type IV when compared with controls. The attachment was found to be decreased on fibronectin and laminin (Figure 2C). This finding is interesting, as collagen type IV is one of the major matrix components found in the basement membrane of normal tissue and organ [21]. We have checked the expressions levels of DJ-1 on various extracellular matrix components after infection with DJ-1 adenovirus using immunoblotting, the expression levels of DJ-1 was found to be similar (Figure S1). Similar experiments were carried out using Sht 290 cells. With this cell line, no significant difference in attachment to collagen type IV, fibronectin or laminin was observed between DJ-1 overexpressing cells and controls (Figure 2D).

### Effect of DJ-1 on endometriotic and endometrial cell proliferation

**Figure 3:**
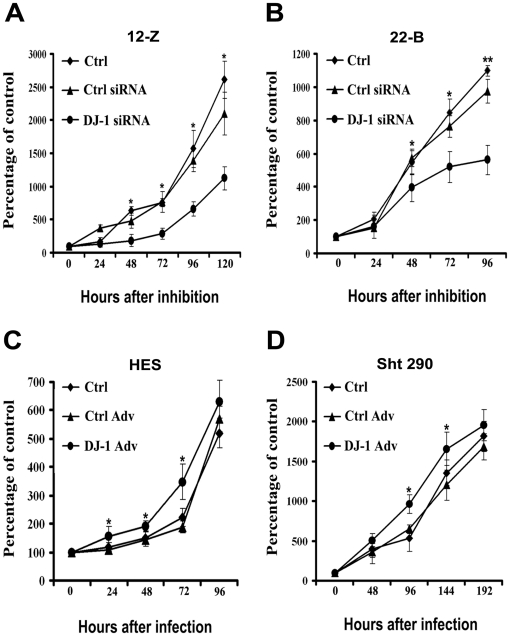
Role of DJ-1 on cell proliferation. Endometriotic epithelial (12-Z) (A) and stromal (22-B) (C) cells were transfected with either DJ-1 siRNA or ctrl siRNA and normal endometrial epithelial (HES) (B) and stromal (Sht 290) (D) cells were infected with control adenovirus (Ctrl Adv) or with DJ-1-GFP adenovirus (DJ-1 Adv), plated on 96 well plate and cell proliferation was determined by MTT assay. Results are expressed as the percentage of control at time 0 h. *Asterisk indicates significant differences (P<0.05) between either DJ-1 knockdown cells or cells overexpressing DJ-1 and controls, as determined by one way ANOVA with post hoc Bonferroni test. Cells which were not transfected with either DJ-1 siRNA or which were not infected with the adenovirus served as controls. Numerical data are expressed as mean ± SE of three independent experiments.

The role of DJ-1 in endometrial and endometriotic cell proliferation was ascertained by using siRNA and overexpression approach. The success of siRNA transfection was 85-90% and DJ-1 gene silencing was upto about 80% in endometriotic cells (Figure S2A and S2B). The cell growth kinetics indicated that down-regulation of DJ-1 by silencing DJ-1 gene decreases endometriotic epithelial and stromal cell proliferation by ∼2.5 and ∼1.5 fold, respectively when compared with controls (Figure 3A and 3B). However, the inhibitory effect was more in endometriotic epithelial cells than in stromal cells. The experiment was also repeated using a second siRNA targeting DJ-1, and similar results were obtained as shown in Figure S2C and S2D.

Further we determined, whether overexpression of DJ-1 affects cell proliferation in normal endometrial cells. Overexpression of DJ-1 in normal endometrial epithelial cells enhances cell proliferation by 1.4 fold within 48 h and it was 1.83 fold more by 72 h when compared with controls (Figure 3C). In endometrial stromal cells, the cell proliferation assay was performed up to 192 h as it is a slow growing cell line. Growth kinetics results showed that overexpression of DJ-1 increases the stromal cell proliferation by 1.5 fold at 96 h (Figure 3D). The expression levels of DJ-1 after infection with DJ-1-GFP adenovirus and control was checked by immunoblotting using GFP antibody (Figure S3A and S3B). It was observed that the expression levels of DJ-1 increases in a time dependent manner.

### DJ-1 plays a role in endometrial cell migration and invasion in endometriosis

**Figure 4:**
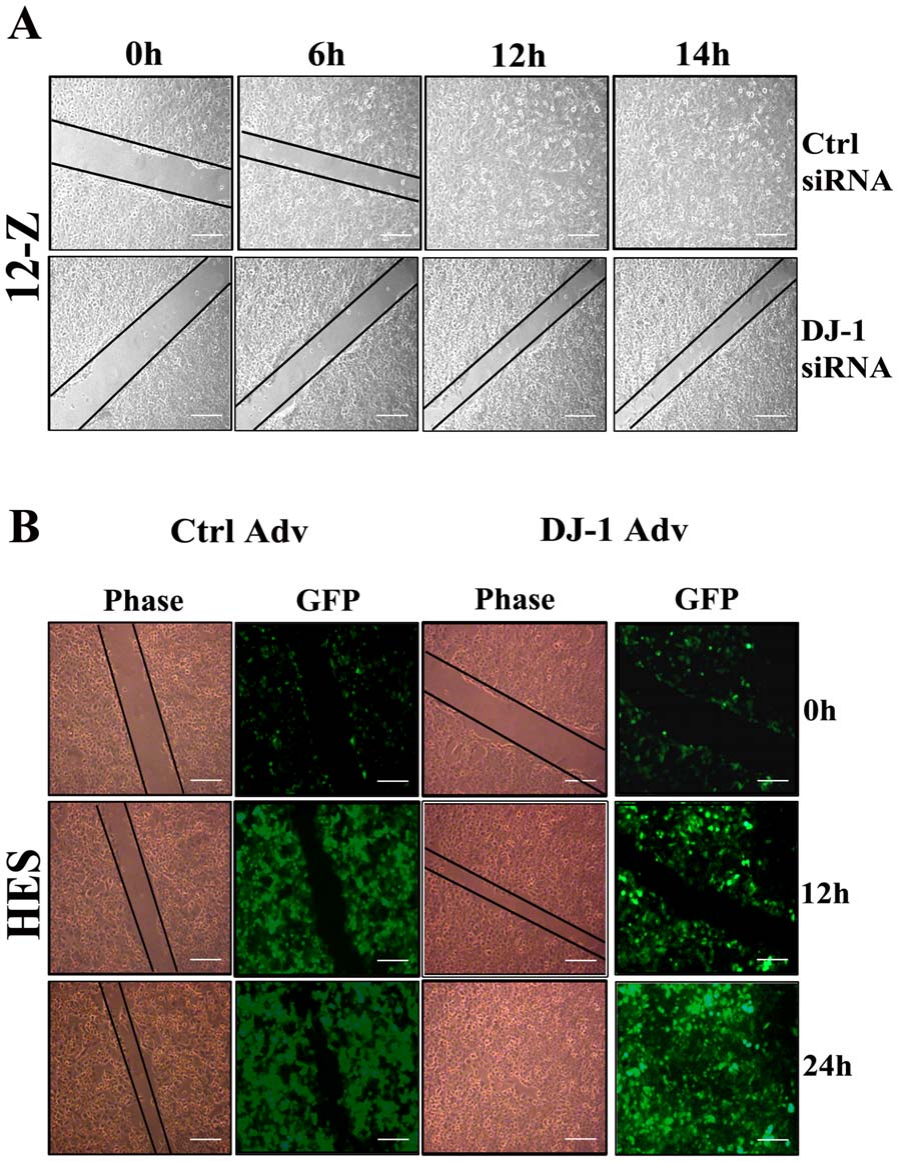
Role of DJ-1 in normal endometrial and endometriotic epithelial cell migration. Inhibition of DJ-1 in endometriotic epithelial cells (12-Z) results in decreased migration (A). Overexpression of DJ-1 in normal endometrial epithelial cells (HES) results in increased migration (B). Cells were either transfected with DJ-1 siRNA, or with control siRNA and 48 h post transfection wound assay was performed. For overexpression, cells were either infected with DJ-1-GFP adenovirus or control adenovirus and 24 h post infection wound assay was performed. Photographs were taken by time lapse microscopy at initial time (0 h) till the termination of the experiments. The experiments were repeated in triplicates.

Wound healing assay was performed to determine the migration capacity of normal endometrial cells and endometriotic cells. In endometriotic epithelial cells (12-Z), the wound was completely healed in 14 h whereas in human endometrial epithelial cells (HES), the wound took more than 24 h (∼36 h) to heal, suggesting that endometriotic epithelial cells migrate faster than normal endometrial epithelial cells (Figure S4A). However, in case of endometriotic (22-B) and normal stromal cells (Sht 290) no significant difference in migration was observed (Figure S4B). To determine whether DJ-1 influences endometriotic and normal endometrial cell migration, and invasion, DJ-1 was knocked down in endometriotic epithelial and stromal cells and then the wound healing assay was performed. Interestingly, it was observed that in endometriotic epithelial cells (12-Z) transfected with ctrl siRNA, the wound healed completely in 14 h but cells in which DJ-1 gene was knocked down, wound healing took more than 14 h (∼24 h) to heal (Figure 4A), suggesting that DJ-1 increases endometriotic epithelial cell migration. In case of endometriotic stromal cells transfected with siRNA no significant difference was observed in migration rate compared to the corresponding control (Figure S5A). The effect of DJ-1 on migration was further confirmed by overexpressing DJ-1 in normal endometrial epithelial and stromal cells by using DJ-1 adenovirus. We observed that, in cells overexpressing DJ-1, wound was completely healed in 24 h as compared to control, in which wound was healed in ∼36 h (Figure 4B). However, overexpression of DJ-1 does not significantly affect migration in normal endometrial stromal cells (Figure S5B).

**Figure 5:**
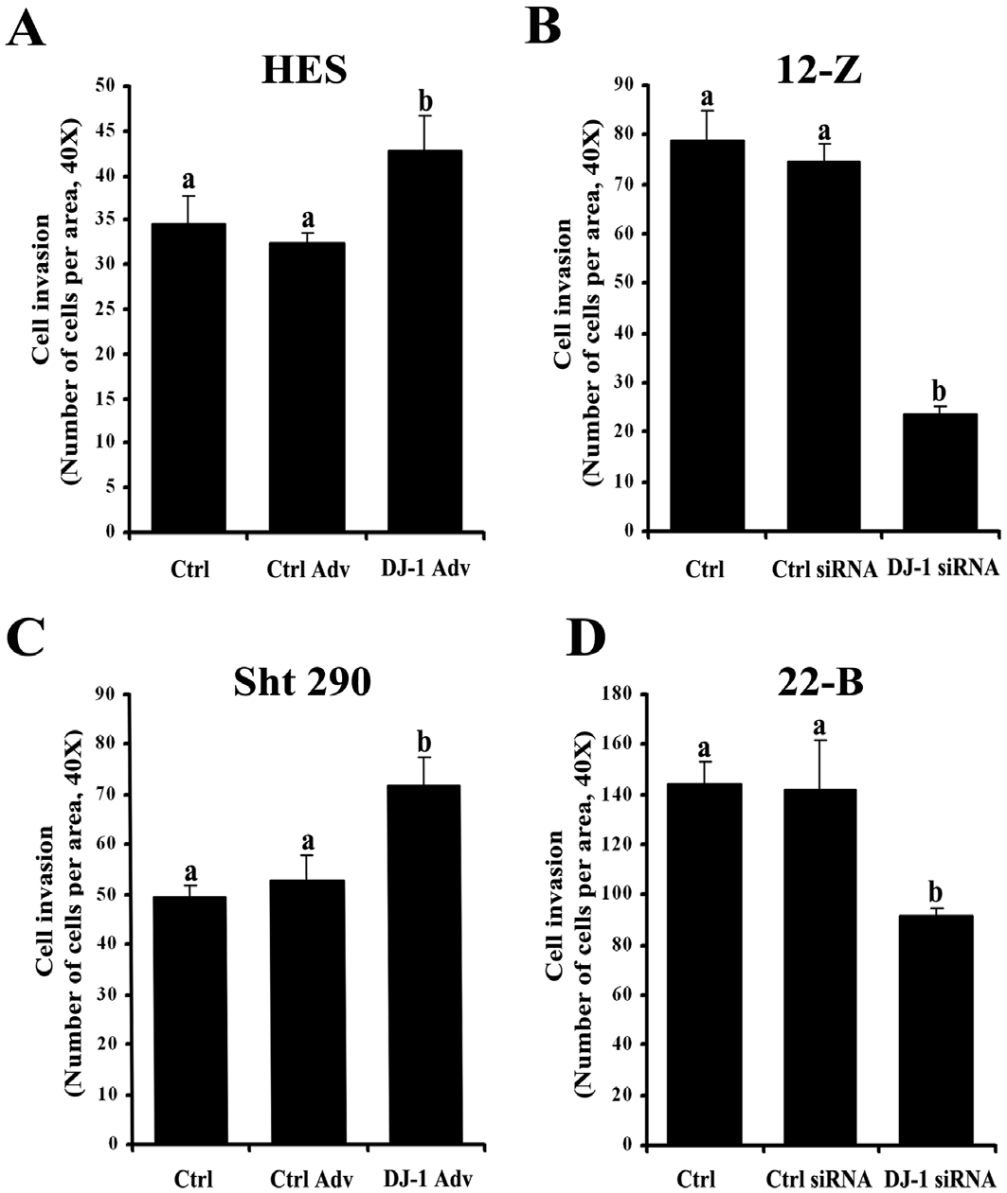
Role of DJ-1 in normal endometrial and endometriotic cell invasion. Normal endometrial epithelial, HES (A) and stromal, Sht 290 (C) cells were infected with control adenovirus (Ctrl Adv) or with DJ-1-GFP adenovirus (DJ-1 Adv) and endometriotic epithelial, 12-Z (B) and stromal, 22-B (D) cells were transfected with either DJ-1 siRNA or ctrl siRNA. At the end of incubation period, matrigel invasion assay was performed. Results are expressed as the number of cells per area at 40X. Bars with different superscripts (a, b) indicate significant differences (p<0.05) between either cells overexpressing DJ-1 or DJ-1 knockdown cells and controls, as determined by one way ANOVA with post hoc Bonferroni test. Cells which were not transfected with either DJ-1 siRNA or which were not infected with the adenovirus served as controls. Numerical data are expressed as mean ± SE of three independent experiments.

Since DJ-1 regulates the process of cell migration, we next determined whether DJ-1 is involved in cell invasion. Results indicated that over-expression of DJ-1 increases the invasion of normal endometrial epithelial and stromal cells by approximately 32 and 35%, respectively (Figure 5A and 5C). Regulation of invasion potential by DJ-1 was further confirmed by knocking down DJ-1 expression using siRNA. Results indicated that silencing of DJ-1 gene decreased invasion of endometriotic epithelial and stromal cells by approximately 68 and 36%, respectively (Figure 5B and 5D).

### Involvement of DJ-1 in PI3K-AKT Pathway

**Figure 6:**
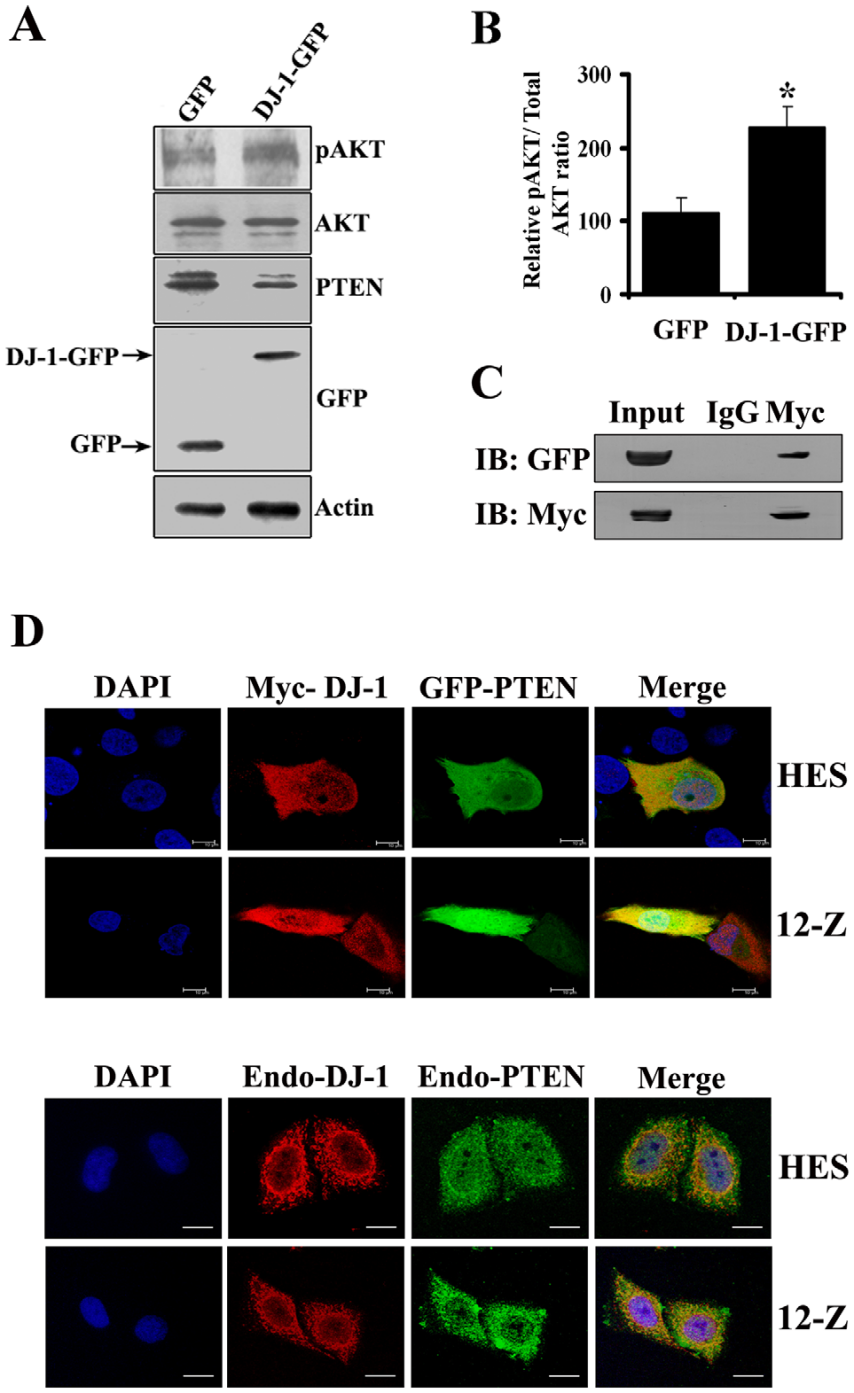
Involvement of DJ-1 in PI3K-AKT pathway. DJ-1 increases the phosphorylation of Akt and downregulates PTEN expression (A). HES cells were transfected with DJ-1-GFP or GFP. After 48 h, transfected cells were collected for immunoblot analysis using anti-pAkt, anti-Akt, anti-PTEN and anti-GFP antibody. Actin was used as an internal control. Densitometry analysis of pAKT to total AKT ratio based on immunoblot analysis in HES cells transfected with DJ-1-GFP or GFP cells (B). *Asterisk indicates significant difference (p<0.05, as determined by Student's t-test) between cases and controls. DJ-1 interacts with PTEN (C). GFP-PTEN was co-immunoprecipitated with Myc-DJ-1. Lysates from HES cells co-transfected with myc-DJ-1 and GFP-PTEN were subjected to IP with anti-myc antibody. Immuoprecipitates and input lysates were analyzed by immunoblotting with anti- Myc and anti-GFP antibodies. Co-localization of DJ-1 with PTEN (D). HES and 12Z cells transfected with GFP-PTEN and myc-DJ-1 were fixed and stained with anti-myc antibody (red). HES and 12Z cells were stained with antibodies against PTEN (green) and DJ-1 (red). The images were visualized with a confocal microscopy.

Recent reports have demonstrated that DJ-1 modulates the PI3K-Akt survival pathway by negatively regulating the function of the tumor suppressor gene PTEN [16]. Therefore, attempts were made to analyze the function of DJ-1 in the PI3K signaling pathway, focusing on the interaction of DJ-1 with PTEN. To investigate, the effect of DJ-1 overexpression on Akt phosphorylation and PTEN expression, HES cells were transiently transfected with DJ-1-GFP or GFP alone. After 48 h, cell lysates were prepared and subjected to immunoblot analysis. In HES cells, overexpression of DJ-1 increases the levels of phosphorylated Akt while the expression level of PTEN was decreased (Figure 6A and 6B). To further determine whether DJ-1 interacts with PTEN, HES and 12-Z cells were co-transfected with GFP-PTEN and myc-DJ-1 and after 48 h immunofluorescence was performed. DJ-1 and PTEN were found to be expressed in cytoplasm and nucleus and confocal analysis showed that DJ-1 colocalizes with PTEN (Figure 6D). Immunofluorescence and confocal studies at endogenous level was also carried out in HES and 12-Z cells by staining for endogenous DJ-1 and PTEN. Consistent with over-expression studies, endogenous DJ-1 colocalizes with endogenous PTEN (Figure 6D). We have also performed immunofluorescence and confocal analysis in Ishikawa cells, which are PTEN negative. The cells were co-transfected with GFP-PTEN and myc-DJ-1. DJ-1 and PTEN were found to be expressed in cytoplasm and nucleus and confocal analysis showed that the extent of colocalization is more in the nucleus than in the cytoplasm (Figure S6). The immunofluorescence studies at endogenous level were also carried out in HeLa cells (human cervical epithelial cancerous cell line) by staining for endogenous DJ-1 and PTEN. Consistent with HES and 12-Z studies, endogenous DJ-1 colocalizes with endogenous PTEN (Figure S6). The interaction was further validated by co-immnoprecipitation assay. HES cells were co-transfected with GFP-PTEN and myc-tagged DJ-1 followed by immunoprecipitation with anti-myc antibody and then immunoblotted with anti-myc and anti-GFP antibodies. The results demonstrated that GFP-PTEN was present in anti-myc precipitated samples (Figure 6C), suggesting that DJ-1 interacts with PTEN. Our results show that DJ-1 interacted with PTEN and its overexpression resulted in decreased expression of PTEN which led to increased phosphorylation of Akt.

## Discussion

In endometriosis, the eutopic endometrium of women shows aberrant expression of many genes/proteins that are involved in regulating various cellular processes [4]-[9]. Recently, we demonstrated that many proteins are differentially expressed in various stages of endometriosis [9]. DJ-1 is one of the proteins that is found to be consistently upregulated in various stages of endometriosis [9]. In the present study, the role for DJ-1 in endometrial cell survival, proliferation, migration, and invasion either by over-expressing DJ-1 in normal endometrial cells or by knocking down DJ-1 expression in endometriotic cells using siRNA has been investigated. The results provide the first evidence that DJ-1 may be involved in the pathogenesis of endometriosis by regulating endometrial cell survival, proliferation, migration, and invasion at ectopic sites.

DJ-1 expression levels were found to be more in endometriotic epithelial and stromal cells than normal endometrial epithelial and stromal cells consistent with the finding of our proteomic analysis [9]. Increased expression of DJ-1 protein in endometriotic cell lines suggests its important role in the pathophysiology of endometriosis. DJ-1 has multiple roles in various biological processes such as cellular transformation [11], protection against oxidative stress mediated apoptosis [18], [19], [20], and transcriptional regulation [18], [22] and in cellular proliferation [15], [23]. Our results demonstrated that overexpression of DJ-1 protects normal endometrial cells against oxidative stress induced cell death, which is in agreement with earlier reports [19], [20].

Endometriosis is characterized by adherence, growth, proliferation, migration, and infiltration of endometrial tissue to the surrounding tissue [2]. It has been demonstrated, using *in vitro* model of the early endometriotic lesion, that endometrial cells attach to mesothelial cells and this process is dependent on the source of endometrial cells [24]. Therefore, firstly we compared the adhesion capacity of normal endometrial and endometriotic cells. It was observed that both the cell types have different adherence capabilities on various ECM components. Therefore, we next determined whether increased DJ-1 protein expression in normal endometrial cells were associated with their increased capacity to attach on various ECM components. Results indicated that normal endometrial epithelial cells overexpressing DJ-1 show different attachment capacity on various ECM components. Interestingly, cells overexpressing DJ-1 attach more on collagen type IV, which is the major ECM component of basement membrane of most tissues and organs in humans [21].

Recent reports have shown that inactivation of DJ-1 by RNA-mediated interference (RNAi) resulted in inhibition of the proliferation in leukemia and laryngeal carcinoma cells [15], [23]. Therefore, we next determined whether DJ-1 regulates normal endometrial and endometriotic cell proliferation. Our result demonstrated that over-expression of DJ-1 significantly increases rate of cell proliferation in normal endometrial epithelial cells as well as in stromal cells. Further, inhibition of DJ-1 expression by siRNA resulted in decreased cell proliferation of endometriotic epithelial and stromal cells. Taken together, these results suggest that DJ-1 is also involved in regulation of endometrial and endometriotic cell proliferation.

Endometriosis is characterized by migration and invasion of endometrial cells at ectopic sites [2]. This led us to investigate the migration ability of normal endometrial and endometriotic cells. We observed that the endometriotic epithelial cells migrate faster than normal endometrial epithelial cells. On the contrary, no differences in rates of migration between normal endometrial and endometriotic stromal cells were observed. This difference might be due to the inherent nature of these cell types. Since the migration rates of normal endometrial and endometriotic cells are different, we next determined whether DJ-1 is associated with endometrial and endometriotic cell migration as well as invasion potential. Results indicated that overexpression of DJ-1 significantly increases the migrating and invading potential of normal endometrial epithelial cells. Further, inhibition of DJ-1 by siRNA significantly decreased the rate of migration and invasion in endometriotic epithelial cells. Interestingly, we observed that neither over-expression of DJ-1 nor inhibition of DJ-1 affects the migration potential of normal endometrial stromal cells and endometriotic stromal cells, respectively. But, overexpression of DJ-1 leads to an increase in the invading potential of normal endometrial stromal cells. Inhibition of DJ-1 resulted in decrease in invasion potential. These observations suggest that DJ-1 regulates migration and invasion in endometrial and endometriotic epithelial cells but only regulates the process of invasion in stromal cell type. This difference might be due to the inherent nature of these two cell types. Such difference between the behavior of endometrial and endometriotic epithelial and stromal cells have been previously reported by Banu et al. [25]. However, the underlined molecular mechanisms for these selective effects are unknown.

In the process of deciphering the role of DJ-1 in endometriosis, we demonstrated that DJ-1 regulates endometrial and endometriotic cell survival, proliferation, migration, and invasion. Further, an attempt has been made to elucidate the molecular mechanisms underlying the DJ-1 dependent cell survival, proliferation, migration, and invasion. Since DJ-1 modulates the PI3K-Akt survival pathway by negatively regulating the function of the tumor suppressor gene PTEN [16], we assessed the expression levels of phosphorylated Akt and PTEN upon DJ-1 over-expression. The results showed that over-expression of DJ-1 increases the levels of phosphorylated Akt but decreases PTEN levels, which is consistent with previous reports [15], [16]. We also demonstrated using colocalization and co-immunoprecipitation assays that DJ-1 interacts with PTEN. DJ-1 inhibits PTEN phosphatase activity through direct interaction of DJ-1 with PTEN in a manner dependent on the oxidative status of C106, resulting in activation of Akt, which promotes cellular transformation [26]. Very recently, Davidson et al. [27], have shown that both the lipid and protein phosphatase activities contribute significantly to the inhibition of both invasive morphology and proliferation. Our study implies that DJ-1 regulates endometrial cell survival, proliferation, migration, and invasion by modulating PI3K-Akt survival pathway by interacting and negatively regulating PTEN. However, the involvement of other mechanisms by which DJ-1 regulates theses cellular processes cannot be ruled out.

In conclusion, in this study we examined the possible role of DJ-1 in the pathogenesis of endometriosis. The results from the present study suggest that DJ-1 protein is highly expressed in endometriotic cells. The increased expression of DJ-1 regulates survival, migration, and invasion of normal endometrial epithelial and stromal cells, and knocking down DJ-1 expression decreases growth, migration, and invasion of endometriotic epithelial and stromal cells, suggesting that DJ-1 might be playing an important role in the pathogenesis of endometriosis.

## Materials and Methods

### Materials

The reagents used in this study were: DMEM, phenol red free DMEM, FCS, and antibiotics (Invitrogen, Carlsbad, CA). PARP antibody (Roche, Mannheim, Germany), GFP antibody (Clontech, Mountain View, CA), actin, phospho-Akt, Akt and PTEN antibodies (Santa Cruz, CA), Caspase-9 antibody (Millipore, Billerica, MA), DJ-1 antibody (MBL International, Woburn, MA), HRP conjugated secondary antibodies (Sigma-Aldrich, St. Louis, MO), Cy3 and Alexa fluor 488 conjugated secondary antibodies (Jackson Immuno Research, Baltimore, PA) and tissue culture dishes and plates (Corning Inc., Corning, NY). The other chemicals used were molecular biological grade from Sigma-Aldrich.

### Cell lines

Immortalized human endometriotic epithelial cells (12-Z) and stromal cells (22-B) [28] and immortalized human endometrial surface epithelial cells (HES) [29] and stromal cells (Sht 290) [30] were used in the study. 12-Z and 22-B cells were a generous gift from Dr. A. Starzinski-Powitz, Institut für Zellbiologie und Neurowissenschaft, Johann Wolfgang Goethe-Universität, Frankfurt, Germany. HES cells were a generous gift from Dr. Asgerally T. Fazleabas, University of Illinois at Chicago, Illinois, USA. Sht 290 cells were a generous gift from David G. Kaufman, University of North Carolina at Chapel Hill, North Carolina, USA. 12-Z and 22-B cells were cultured in DMEM containing 10% FCS and HES and Sht 290 cells were maintained in phenol red free DMEM containing 10% and 2% charcoal treated FCS, respectively. The media also contained penicillin (100 U/ml) and streptomycin (100 g/ml). Cells were maintained in a humidified atmosphere of 5% CO_2_ in air at 37°C.

### Small interfering RNA

Cells were transfected with 200 nM DJ-1 SMARTpool siRNA that specifically targets the DJ-1 gene or non silencing control using Lipofectamine-2000 according to manufacturer's instructions. An individual DJ-1 siRNA is also used. SiRNA duplexes were obtained from Dharmacon, Inc. (Lafayette, CO).

### Generation of adenoviral vectors

Adenoviral vector for expressing DJ-1-GFP fusion protein was prepared as described using pAdEASY system [31].

### Protein extraction and immunoblotting

Cells transfected with pEGFP-DJ-1 or pEGFP vectors (treated or untreated) were lysed .The proteins were eluted by boiling in 3X SDS sample buffer and resolved by SDS-PAGE. Protein concentration was determined using amido black method [32]. The proteins were transferred to nitrocellulose membrane for immunoblot analysis as previously described [9]. Primary antibodies for DJ-1 (1∶1000), PARP (1∶3000), GFP (1∶ 1000), caspase 9 (1∶1000), pAKT (1∶1000), AKT (1∶1000), PTEN (1∶250) or actin (1∶750) were used. The secondary antibody used was conjugated to horseradish peroxidase (HRP) (1∶10 000). The blots were developed using the ECL kit (Millipore, Billerica, MA). Exposed films were scanned and bands of interest were quantified using Gene-Tools version 3.06.04 from SynGene (Cambridge, U.K.).

### Cell Adhesion Assay

The cell adhesion assay was done as described [33]. Briefly, cells were either infected with DJ-1-GFP adenovirus and control GFP adenovirus for 24 h. Cell suspension (1×10^4^ cells) was added to the wells of a 96-well microtitre plate coated with collagen type IV, fibronectin, laminin or poly-L-lysine and incubated for 20 min. At the end of incubation, MTT assay was performed. Cells treated with poly-L-lysine served as positive control. Data is expressed as the mean ± SE of three independent experiments.

### Cell Proliferation Assay

Cells were either infected with DJ-1-GFP adenovirus or control GFP adenovirus for 24 h, or transfected with DJ-1 siRNA or control siRNA for 48 h. 100 µl of cell suspension (1×10^3^ cells) was plated in a 96-well microtitre plate in triplicates. At the end of 0, 24, 48, and 96 h, MTT assay was performed. Data is expressed as the mean ± SE of three independent experiments.

### Wound Healing Assay

The wound assay was done as described [34]. Briefly, cells were either infected with DJ-1-GFP adenovirus or control GFP adenovirus for 24 h, or transfected with DJ-1 siRNA or control siRNA for 48 h. At the end of incubation a wound was created manually. The wound was then visualized under an automated time-lapse microscope equipped with a temperature control chamber. The images of the same region were acquired automatically for 24–36 h.

### Matrigel Invasion Assay

In vitro invasion assay was performed using Matrigel-coated 24-well chambers according to the manufacturer's instructions (BD Bioscience, Bedford, MA) and as described [25]. Briefly, the cells were either infected with DJ-1-GFP adenovirus or control GFP adenovirus for 24 h, or transfected with DJ-1 siRNA or control siRNA for 48 h. After rehydration of the chambers, the cells (5×10^4^ cells per chamber) in 500 µl growth medium were seeded onto the upper chamber. In the lower chamber, 500 µl DMEM plus 10% FCS was placed. Invasion of cells was measured as number of cells invaded from a defined area of the Matrigel-coated microfilter through micropores in 24 h. The micropore filters were stained with haematoxylin and eosin and cells that migrated through the filters were counted in eight representative areas under an inverted microscope (Axioplan 2) at 400× magnification and expressed as the mean ± SE of three independent experiments.

### Confocal microscopy

Cells grown on coverslips were transfected with myc-DJ-1 and GFP-PTEN plasmids. Cells were fixed and stained with antibody against myc. For endogenous staining, HES and 12-Z cells were fixed and stained with antibodies against DJ-1 and PTEN. Cells were then stained with flurophores conjugated secondary antibodies and mounted in Vectashield mounting media containing DAPI (Burlingame, CA). The cells were analyzed for colocalization using a LSM 510 Meta confocal microscope (Carl Zeiss Microimaging, Jena). Serial optical sections in the Z-axis of the cells were collected at 0.33 µm intervals with a 63× oil immersion objective lens (NA 1.4). Quantitative analysis of colocalization was carried out by using LSM 510 software.

### Co-immunoprecipitation

HES cells transfected with the myc-DJ-1 and GFP-PTEN plasmids for 48 h, were lysed at 4°C for 20 minutes in a buffer containing 25 mM Tris-HCl, pH 7.4, 150 mM NaCl, 1.0% Triton X-100, 1 mM PMSF, 0.1% BSA, 5 mM EDTA and protease inhibitor cocktail (Roche). Lysates were centrifuged at 10,000 g for 10 minutes at 4°C and the supernatant was used for immunoprecipitation using 2 µg of c-myc rabbit polyclonal antibody or 2 µg of normal rabbit IgG as control antibody. Complexes were precipitated using A/G plus agarose beads (Santa Cruz), washed and lysed in 20 µl of 3X SDS sample buffer. The samples were resolved on 10% SDS-PAGE and subjected to immunoblot analysis as described above.

### Statistical analysis

Significant differences in the data were determined by one-way and two-way analysis of variance (ANOVA) and Student's t-test (PRISM, version 3). Post hoc comparisons were made using Bonferroni test. P values of <0.05 were considered significant.

## Supporting Information

Figure S1 **Expression of DJ-1 on various extracellular matrix components.** Immunoblot analysis showing the expression levels of DJ-1 on various extracellular matrix components after infection with DJ-1-GFP adenovirus using GFP antibody. Cells which were plated on uncoated wells served as control.
(TIF)

Figure S2 **Effect of DJ-1 and control siRNA on DJ-1 expression and endometriotic cell proliferation.** Effect of DJ-1 siRNA on expression of DJ-1 protein after 48, 72, 96, 120, and 144 h post transfection (A). Effect of ctrl siRNA on expression of DJ-1 protein after 48, 72, 96, 120, and 144 h post transfection (B). Cells which were not transfected with either DJ-1 siRNA or control siRNA served as control. Endometriotic epithelial (12-Z) (C) and stromal (22-B) (D) cells were transfected with either DJ-1 siRNA or ctrl siRNA and plated on 96 well plates. Cell proliferation was determined by MTT assay. Results are expressed as the percentage of control at time 0 h. *Asterisk indicates significant differences (P<0.05) between DJ-1 knockdown cells and controls, as determined by one way ANOVA with post hoc Bonferroni test. Cells which were not transfected with either DJ-1 siRNA or control siRNA served as control. Numerical data are expressed as mean ± SE of three independent experiments.
(TIF)

Figure S3 **Expression of DJ-1 after infection with DJ-1-GFP and control adenovirus.** Immunoblot analysis showing the expression levels of DJ-1 on infection with either DJ-1-GFP adenovirus (A) or GFP control adenovirus (C) using GFP antibody after 24, 48, 72, 96, and 120 h after infection. Cells which were transfected with either DJ-1-GFP or GFP control plasmid served as control. B and D represent densitometry analysis of DJ-1-GFP and GFP protein to actin ratio based on immunoblot analysis, respectively.
(TIF)

Figure S4 **Wound healing assay to determine migration in normal and endometriotic cells.** Panels in A show the temporal sequence (0, 12 and 24 h) of wound healing in normal endometrial epithelial (HES) and endometriotic epithelial (12-Z) cells. Panels in B show the temporal sequence (0, 12 and 24 h) of wound healing in normal endometrial stromal (Sht 290) and endometriotic stromal (22-B) cells. Photographs were taken by time lapse microscopy at initial time (0 h) till the termination of the experiments. Experiments were repeated in triplicates.
(TIF)

Figure S5 **Role of DJ-1 in normal endometrial and endometriotic stromal cell migration.** Inhibition of DJ-1 in endometriotic stromal cells (22-B) does not significantly affect migration (A). Overexpression of DJ-1 in normal endometrial stromal cells (Sht 290) does not significantly affect migration (B). Cells were either transfected with DJ-1 siRNA, or with control siRNA and 48h post transfection wound assay was performed. For overexpression, cells were either infected with DJ-1-GFP adenovirus, or with control adenovirus and 24h post infection wound assay was performed. Wound photographs were taken by time lapse microscopy at initial time (0 h) till the termination of the experiments. The experiments were repeated in triplicates.
(TIF)

Figure S6 **Co-localization of DJ-1 with PTEN in Ishikawa and HeLa cells.** Ishikawa cells transfected with GFP-PTEN and myc- DJ-1 were fixed and stained with anti-myc antibody (red). HeLa cells were stained with antibodies against PTEN (green) and DJ-1 (red).The images were visualized with a confocal microscopy.
(TIF)
