# Supplementary figures and images for: The Role of DJ-1 in the Pathogenesis of Endometriosis
Source: PLoS One. 2011 Mar 21;6(3):e18074. doi: 10.1371/journal.pone.0018074 (PMC3061880; doi:10.1371/journal.pone.0018074)

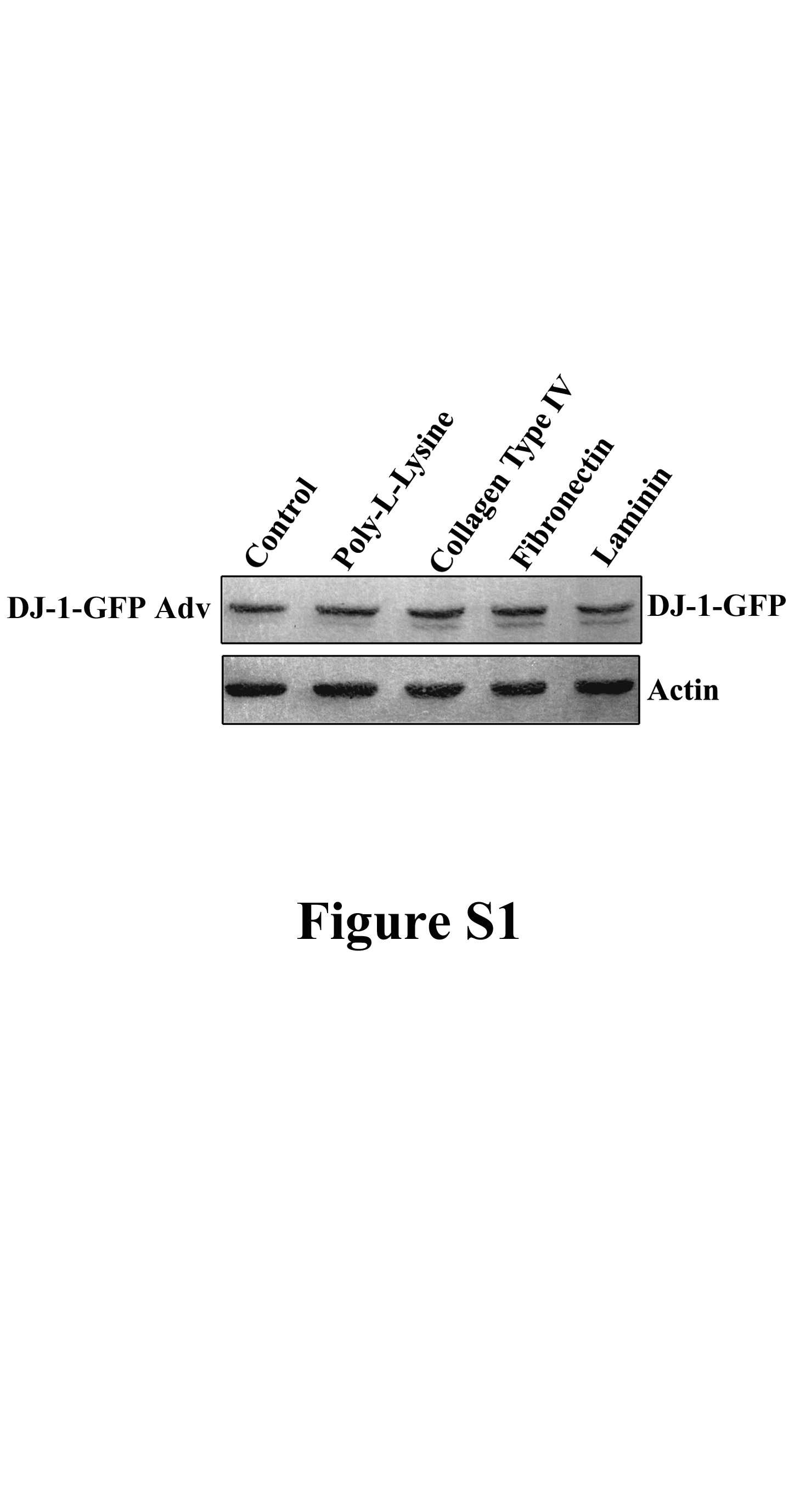

Supplement: Figure S1 — Expression of DJ-1 on various extracellular matrix components. Immunoblot analysis showing the expression levels of DJ-1 on various extracellular matrix components after infection with DJ-1-GFP adenovirus using GFP antibody. Cells which were plated on uncoated wells served as control. (TIF) [file pone.0018074.s001.tif]

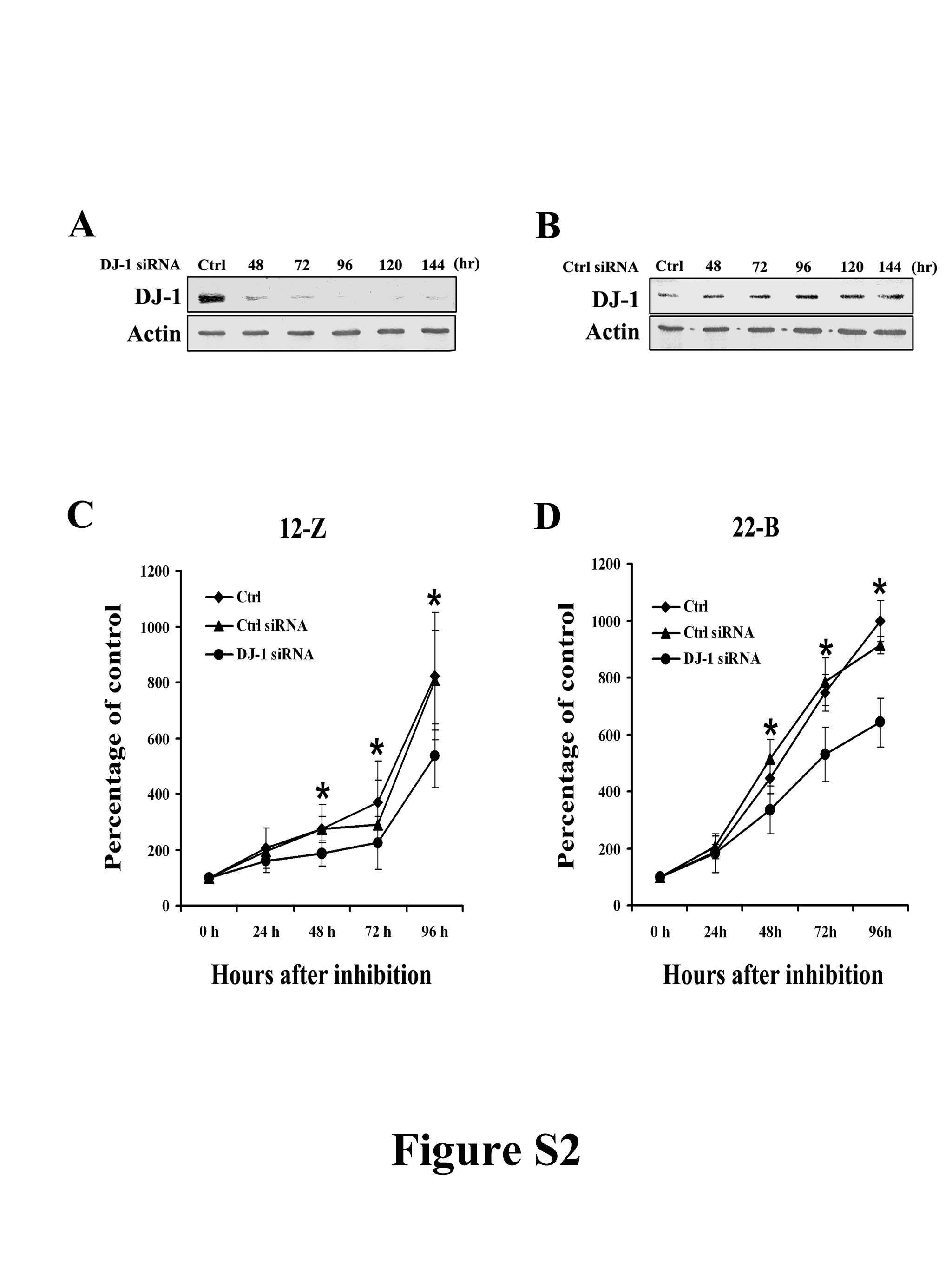

Supplement: Figure S2 — Effect of DJ-1 and control siRNA on DJ-1 expression and endometriotic cell proliferation. Effect of DJ-1 siRNA on expression of DJ-1 protein after 48, 72, 96, 120, and 144 h post transfection (A). Effect of ctrl siRNA on expression of DJ-1 protein after 48, 72, 96, 120, and 144 h post transfection (B). Cells which were not transfected with either DJ-1 siRNA or control siRNA served as control. Endometriotic epithelial (12-Z) (C) and stromal (22-B) (D) cells were transfected with either DJ-1 siRNA or ctrl siRNA and plated on 96 well plates. Cell proliferation was determined by MTT assay. Results are expressed as the percentage of control at time 0 h. *Asterisk indicates significant differences (P<0.05) between DJ-1 knockdown cells and controls, as determined by one way ANOVA with post hoc Bonferroni test. Cells which were not transfected with either DJ-1 siRNA or control siRNA served as control. Numerical data are expressed as mean ± SE of three independent experiments. (TIF) [file pone.0018074.s002.tif]

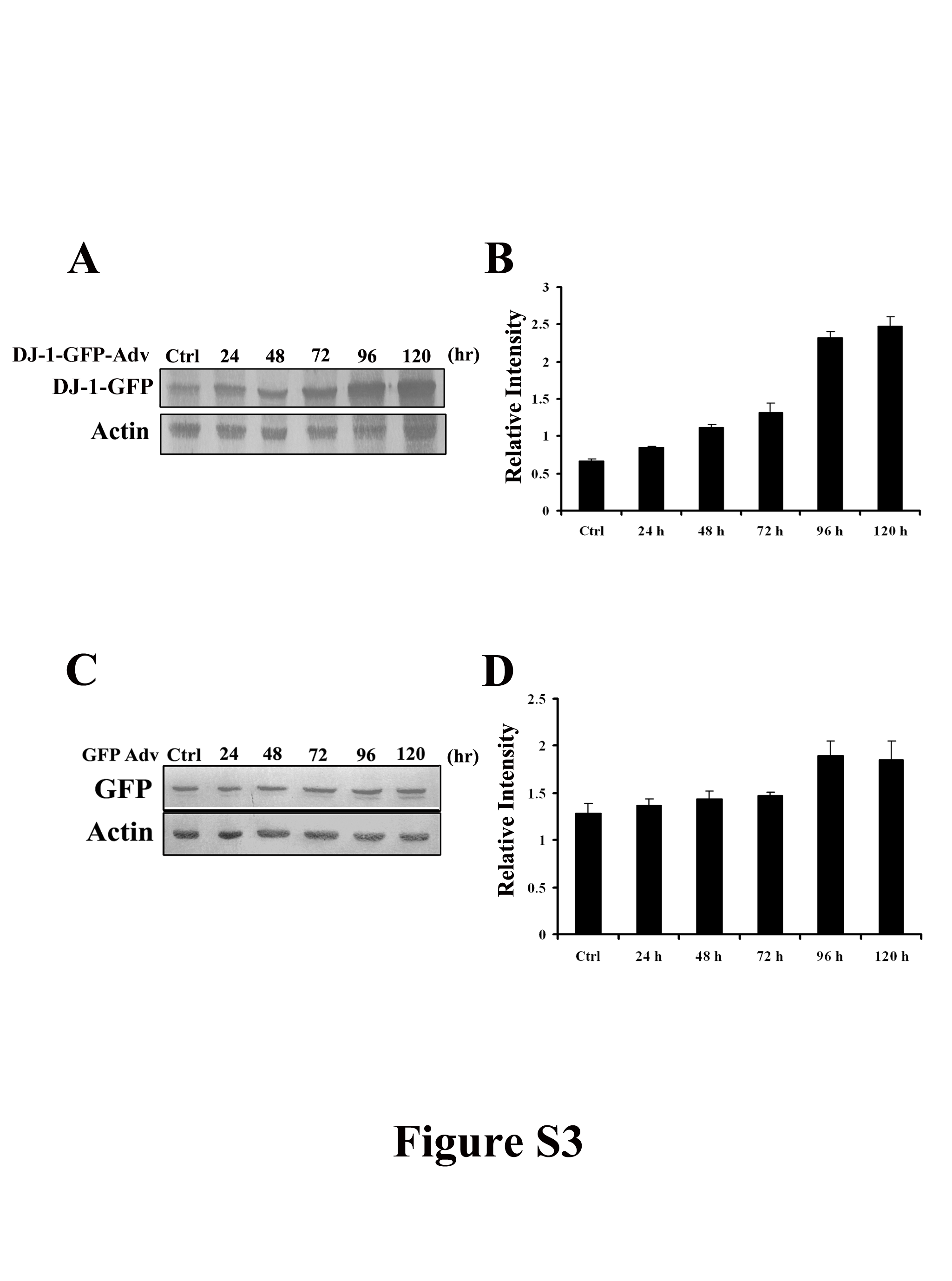

Supplement: Figure S3 — Expression of DJ-1 after infection with DJ-1-GFP and control adenovirus. Immunoblot analysis showing the expression levels of DJ-1 on infection with either DJ-1-GFP adenovirus (A) or GFP control adenovirus (C) using GFP antibody after 24, 48, 72, 96, and 120 h after infection. Cells which were transfected with either DJ-1-GFP or GFP control plasmid served as control. B and D represent densitometry analysis of DJ-1-GFP and GFP protein to actin ratio based on immunoblot analysis, respectively. (TIF) [file pone.0018074.s003.tif]

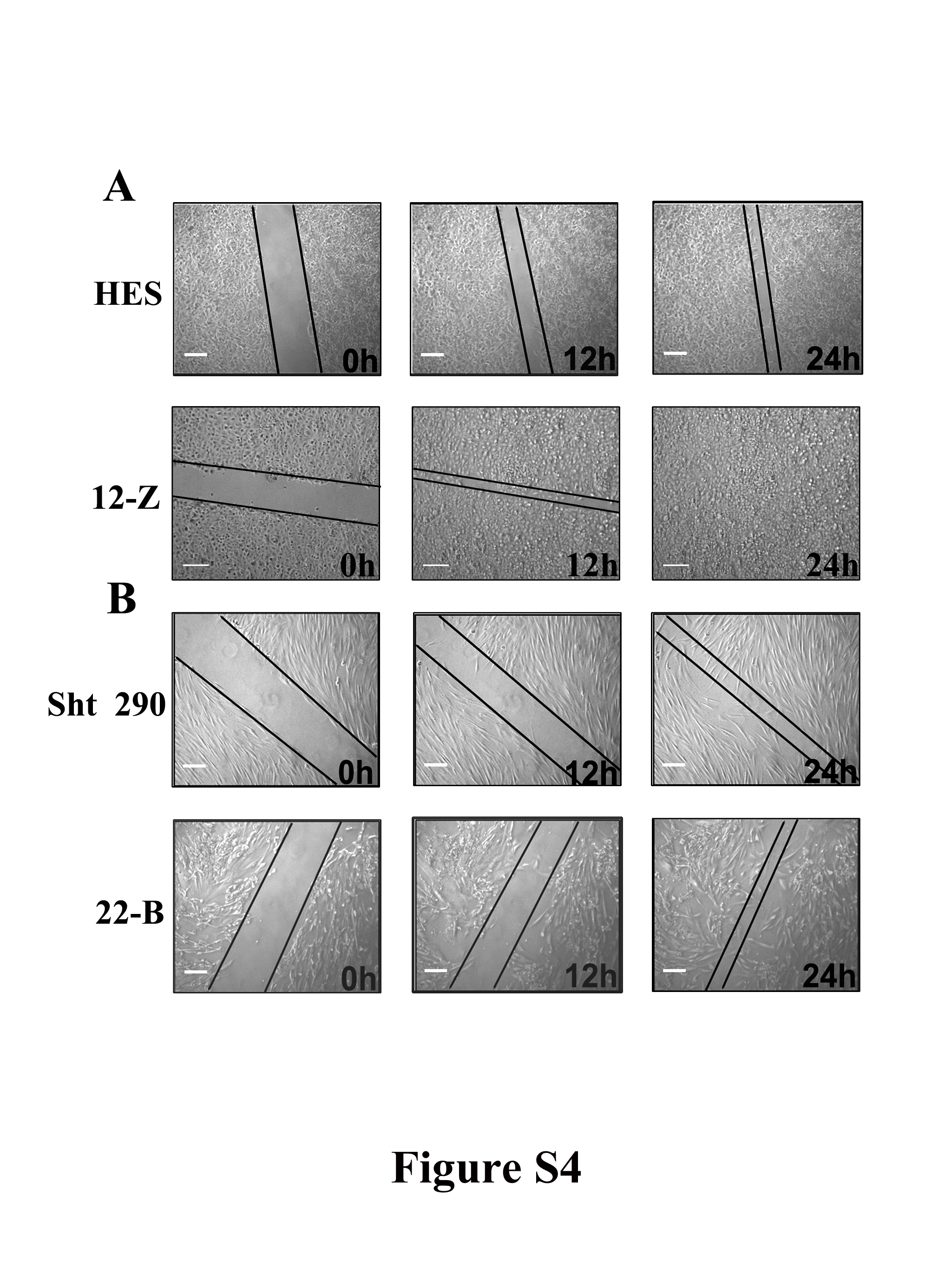

Supplement: Figure S4 — Wound healing assay to determine migration in normal and endometriotic cells. Panels in A show the temporal sequence (0, 12 and 24 h) of wound healing in normal endometrial epithelial (HES) and endometriotic epithelial (12-Z) cells. Panels in B show the temporal sequence (0, 12 and 24 h) of wound healing in normal endometrial stromal (Sht 290) and endometriotic stromal (22-B) cells. Photographs were taken by time lapse microscopy at initial time (0 h) till the termination of the experiments. Experiments were repeated in triplicates. (TIF) [file pone.0018074.s004.tif]

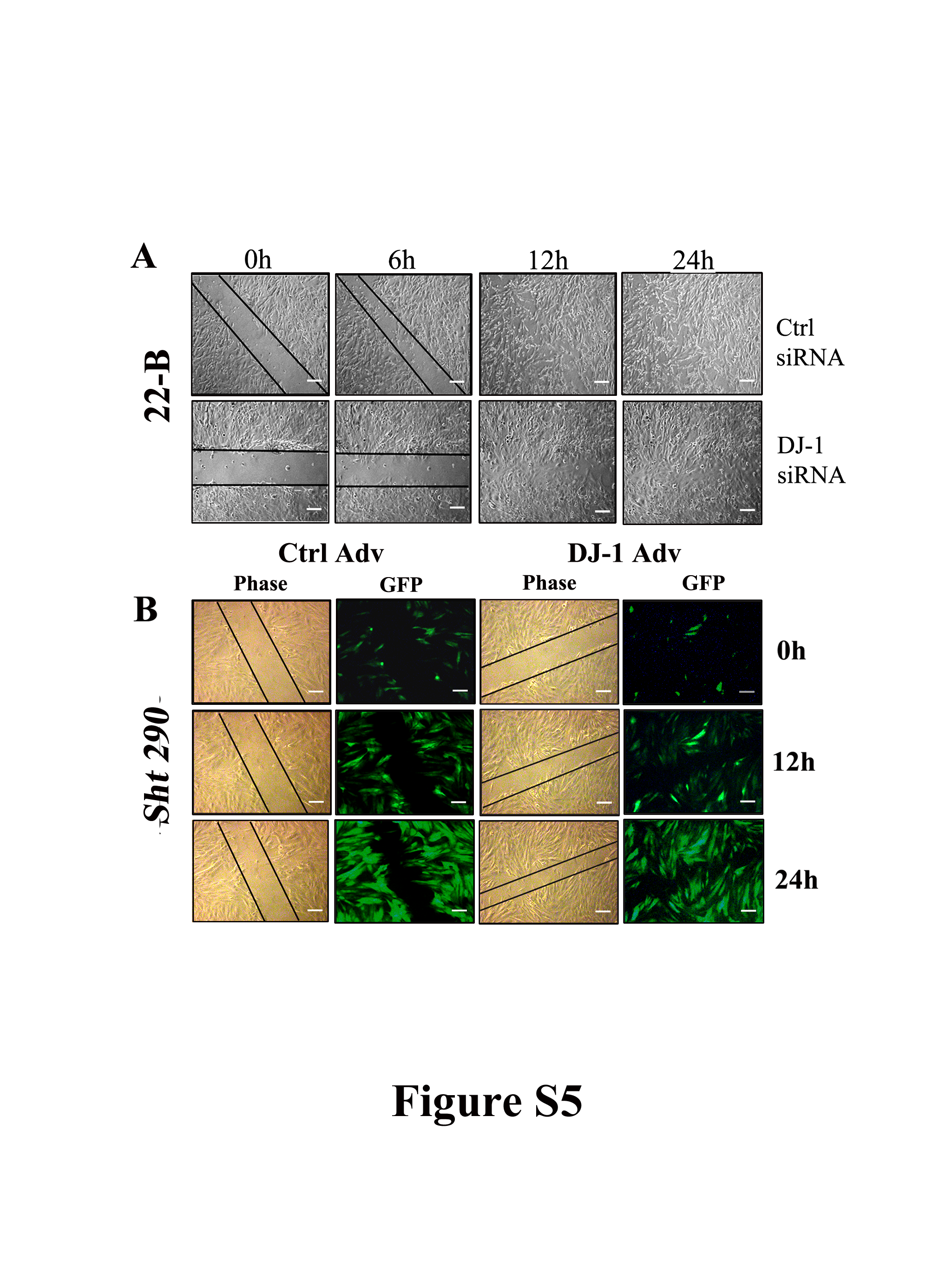

Supplement: Figure S5 — Role of DJ-1 in normal endometrial and endometriotic stromal cell migration. Inhibition of DJ-1 in endometriotic stromal cells (22-B) does not significantly affect migration (A). Overexpression of DJ-1 in normal endometrial stromal cells (Sht 290) does not significantly affect migration (B). Cells were either transfected with DJ-1 siRNA, or with control siRNA and 48h post transfection wound assay was performed. For overexpression, cells were either infected with DJ-1-GFP adenovirus, or with control adenovirus and 24h post infection wound assay was performed. Wound photographs were taken by time lapse microscopy at initial time (0 h) till the termination of the experiments. The experiments were repeated in triplicates. (TIF) [file pone.0018074.s005.tif]

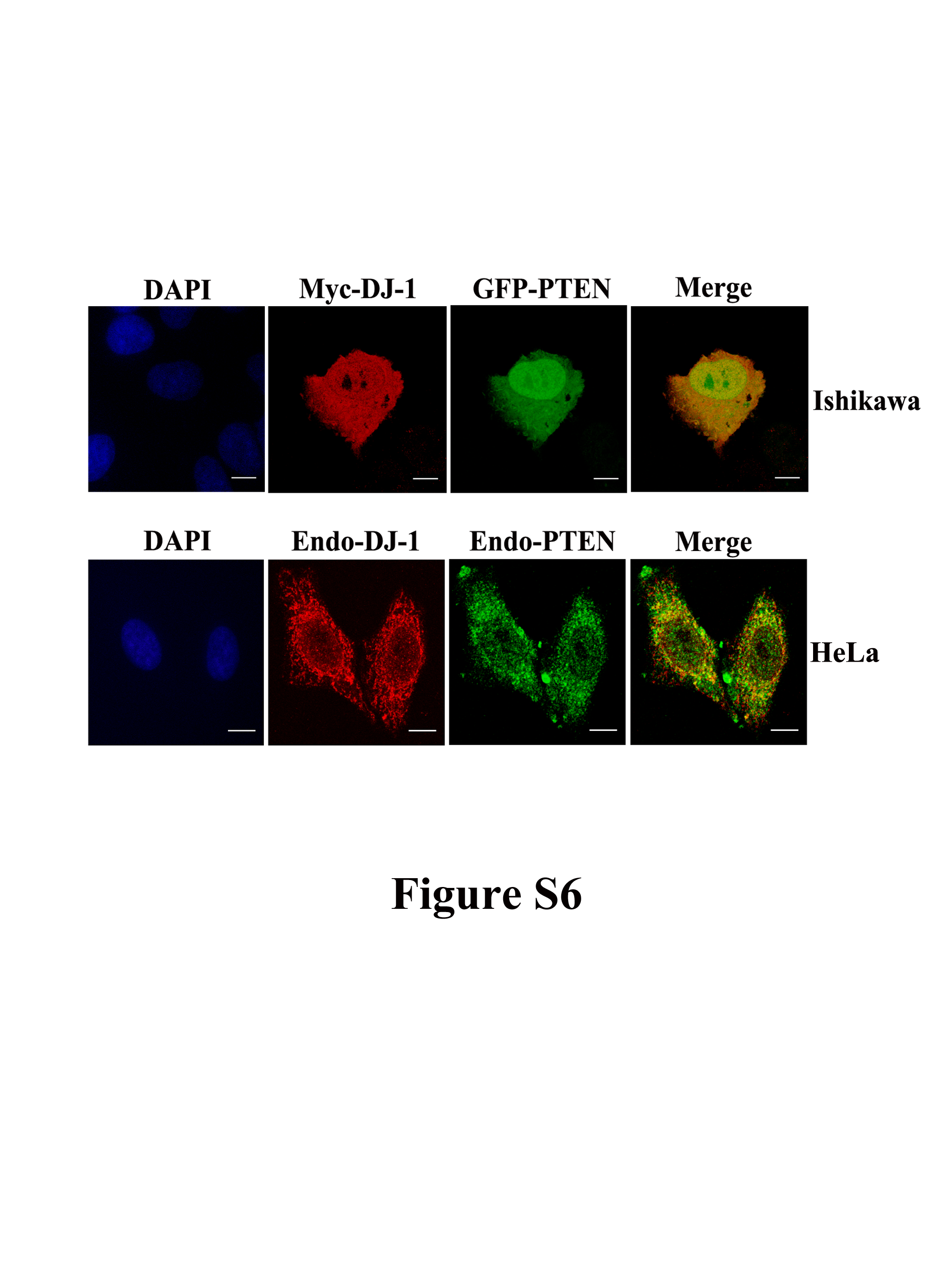

Supplement: Figure S6 — Co-localization of DJ-1 with PTEN in Ishikawa and HeLa cells. Ishikawa cells transfected with GFP-PTEN and myc- DJ-1 were fixed and stained with anti-myc antibody (red). HeLa cells were stained with antibodies against PTEN (green) and DJ-1 (red).The images were visualized with a confocal microscopy. (TIF) [file pone.0018074.s006.tif]
